# Aflibercept Off-Target Effects in Diabetic Macular Edema: An In Silico Modeling Approach

**DOI:** 10.3390/ijms25073621

**Published:** 2024-03-23

**Authors:** Morgane Blanot, Ricardo Pedro Casaroli-Marano, Jordi Mondéjar-Medrano, Thaïs Sallén, Esther Ramírez, Cristina Segú-Vergés, Laura Artigas

**Affiliations:** 1 Anaxomics Biotech S.L., 08007 Barcelona, Spain; morblanot@hotmail.com (M.B.); esther.ramirez@anaxomics.com (E.R.); cristina.segu@anaxomics.com (C.S.-V.); laura.artigas@anaxomics.com (L.A.); 2 Department of Surgery (FMCS), Universitat de Barcelona, 08007 Barcelona, Spain; 3 Hospital Clínic de Barcelona (IDIBAPS), Universitat de Barcelona, 08007 Barcelona, Spain; 4 Bayer Hispania S.L., 08970 Sant Joan Despí, Spain; jordi.mondejar@bayer.com (J.M.-M.); thais.sallen@bayer.com (T.S.); 5 Research Programme on Biomedical Informatics (GRIB), Departament de Ciències Experimentals i de la Salut, Universitat Pompeu Fabra, 08002 Barcelona, Spain

**Keywords:** intravitreal aflibercept injection, systems biology, vascular endothelial growth factor, inflammation, oxidative stress, angiogenesis, blood–retinal barrier permeability, TPMS, machine learning

## Abstract

Intravitreal aflibercept injection (IAI) is a treatment for diabetic macular edema (DME), but its mechanism of action (MoA) has not been completely elucidated. Here, we aimed to explore IAI’s MoA and its multi-target nature in DME pathophysiology with an in silico (computer simulation) disease model. We used the Therapeutic Performance Mapping System (Anaxomics Biotech property) to generate mathematical models based on the available scientific knowledge at the time of the study, describing the relationship between the modulation of vascular endothelial growth factor receptors (VEGFRs) by IAI and DME pathophysiological processes. We also undertook an enrichment analysis to explore the processes modulated by IAI, visualized the effectors’ predicted protein activity, and specifically evaluated the role of VEGFR1 pathway inhibition on DME treatment. The models simulated the potential pathophysiology of DME and the likely IAI’s MoA by inhibiting VEGFR1 and VEGFR2 signaling. The action of IAI through both signaling pathways modulated the identified pathophysiological processes associated with DME, with the strongest effects in angiogenesis, blood–retinal barrier alteration and permeability, and inflammation. VEGFR1 inhibition was essential to modulate inflammatory protein effectors. Given the role of VEGFR1 signaling on the modulation of inflammatory-related pathways, IAI may offer therapeutic advantages for DME through sustained VEGFR1 pathway inhibition.

## 1. Introduction

Diabetic macular edema (DME) is a serious eye complication of diabetes mellitus and one of the major causes of loss of vision and blindness. The prevalence of DME is 4.2–7.9% in diabetes mellitus type 1 and 1.4–12.8% in diabetes mellitus type 2 [1]. The etiology of the disease is complex, but hyperglycemia is considered its major pathological factor; high levels of blood sugar lead to damaged blood vessels in the eye that leak fluid into the macula [2]. DME is characterized by a thickening of the macular area caused by chronic damage to the retinal neurovascular unit [3]. This disease is multifactorial, resulting from the involvement of several pathophysiological mechanisms related to angiogenesis and altered permeability of the blood–retinal barrier (BRB). However, recent investigations have also pointed toward the important role of inflammation in the development of DME [4]. In the context of diabetic retinopathy, angiogenesis can be triggered by retinal hypoxia, oxidative and inflammatory stress, and vessel permeability or breakage [5]. This can alter the balance between angiostatic and angiogenic factors, leading to an increase in pro-angiogenic factors that impact the retina and cause microvasculature impairment, an altered vascular network, microaneurysms, increased leakage, and edema [5]. The proteins involved in these processes include members of the vascular endothelial growth factor (VEGF) family, Galectin-1 (Gal-1) and their receptors, and other angiogenic proteins, such as neuropilin-1 (NRP1), erythropoietin (EPO) receptor, angiopoietin-2 (ANGPT2), platelet-derived growth factor subunit B (PDGFB), and fibroblast growth factor 2 (FGF2) [6]. Several members of the VEGF family have been identified as key angiogenic and vascular permeability factors upregulated in ischemic retinopathies [7]. The VEGF family of growth factors consists of several homologs, such as VEGF-A, VEGF-B, and placental growth factor (PlGF). The latter is a homologous factor to VEGF-A and has been shown to be involved in angiogenesis-dependent retinal disorders [8]. Non-lymphatic VEGF family signaling is mediated by VEGF receptor 1 (VEGFR1) and VEGF receptor 2 (VEGFR2), proteins with tyrosine kinase activity. VEGF-A is a common ligand for both, although it primarily signals through VEGFR2 and shows weaker signaling activity through VEGFR1 [6]. VEGF-A is one of the primary targets and is the most widely used in intravitreal agents treating retinopathies [9]. Other ligands involved in VEGFR signaling are PlGF and VEGF-B, which bind to VEGFR1 and Gal-1, and have been reported to promote VEGFR2 phosphorylation and subsequent angiogenic function [10,11]. PlGF and VEGF-B have also been shown to bind NRP1 on endothelial cells, promoting the NRP1/VEGFR2-mediated signaling pathway and NRP1-mediated biological activities [12]. Additionally, VEGFR1 and VEGFR2 pathways crosstalk, which adds more complexity to VEGF signaling [13]. While VEGFR2 was previously considered to be the primary receptor implicated in VEGFs’ permeability and angiogenic activities, emerging evidence suggests that VEGFR1 signaling also plays a crucial role in pathological conditions [13] since it plays a notable role in the regulation of inflammatory mechanisms [14].

Treatments for DME may include laser photocoagulation, surgery by pars plana vitrectomy, and intravitreal therapy using steroids or anti-VEGF agents [15]. Given the importance of VEGF in DME pathophysiology, anti-VEGF agents are considered the first-line treatment for center-involving DME [16]. The intravitreal aflibercept injection (IAI) contains a fully human recombinant fusion protein, which includes the second domain of human VEGFR1 and the third domain of human VEGFR2 [17]. This drug was first approved for neovascular age-related macular degeneration and later for other retinal vascular pathologies (DME, myopic choroidal neovascularization, and macular edema secondary to retinal vein occlusion) by the European Medicines Agency (EMA) and US Food and Drug Administration (FDA) [18]. Its mechanism of action (MoA) consists of the inhibition of the soluble ligands VEGF-B, PlGF, VEGF-A, and Gal-1 with a specific affinity greater than that of VEGFRs [16,17,18]. Therefore, IAI indirectly blocks VEGFR1 and VEGFR2 signaling pathways [16,19], which results in a multi-target mechanistic profile, whereas other intravitreal anti-VEGF agents only target the VEGF-A/VEGFR2 axis [16,19].

Several randomized clinical trials have demonstrated the efficacy and safety of IAI in DME treatment [20,21,22]. However, its MoA, beyond the direct effect upon its targets and its impact on the downstream pathways to modulate DME pathophysiology, has not been completely elucidated [23]. Different preclinical studies have shown that IAI exerts an effect on inflammation and BRB alteration [24,25], suggesting a more complex role in the DME pathophysiology, probably linked to its capacity to modulate the VEGFR1 pathway.

In silico modeling has previously been used to unravel the MoA of multifactorial pathologies and their treatments [26,27,28]. Here, we report the use of a systems biology modeling approach, based on a previously applied algorithm similar to a Multilayer Perceptron of an Artificial Neural Network over the human protein–protein interaction network (where proteins are represented as neurons and the edges of the network are used to transfer the information) [29,30,31]. The bibliography-based information regarding DME and IAI, compiled through a meticulous manual curation of related scientific literature and following our protocol described in the methodology section, was used to identify key proteins to focus the modeling and analysis in the specific section of the human protein–protein interaction map. The constructed models were used to calculate how the signal flows through the network in different scenarios and thereby calculate the predicted protein activity with the objective of exploring the MoA of IAI and its multi-target nature in the pathophysiology of DME beyond its anti-angiogenic effects.

## 2. Results

### 2.1. DME Interactome Generation and IAI-Treated DME General Model Evaluation

**Figure 1:**
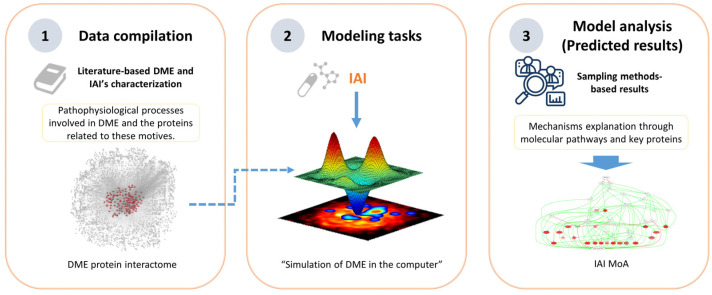
Overview of the Therapeutic Performance Mapping System (TPMS) modeling technique, proprietary to Anaxomics, that integrates (1) data compilation of available biological, pharmacological, and medical knowledge to define diabetic macular edema (DME); (2) mathematical model generation using the validated top-down systems biology- and machine learning-based TPMS approach; and (3) model analyses employing model outputs to define the mechanism of action (MoA) of intravitreal aflibercept injection (IAI).

We obtained computational models of the effect of IAI on DME by employing the Therapeutic Performance Mapping System (TPMS, Anaxomics Biotech, Barcelona, Spain) technology, a systems-biology-based machine learning approach that creates multiple models to understand the MoA of a drug [30,32]. In this particular scenario, the TPMS was based on the compilation of bibliographically based available information to identify key proteins to center the modeling and analysis in the region of interest of the human protein–protein interaction map. Then, the TPMS provided the platform for modeling IAI mechanisms over DME through the human protein–protein interaction map using general human drug–pathophysiology relationships (training set) and for modeling solutions analysis to explore the multi-target MoA of IAI (Figure 1).

Although the TPMS models are protein-based (UniProtKB/Swiss-Prot entries are used as basic units of the protein network), the interactome in which they are built includes gene and RNA regulation data. Thus, for standardization purposes, we will use gene names to refer to all genes/proteins of model results mentioned in tables and figures. Further information on the genes’/proteins’ nomenclature is provided in Supplementary Table S1 to facilitate the reading.

The compilation of bibliography-based information regarding DME led to the identification of four pathophysiological processes (oxidative stress, inflammation, BRB alteration, and angiogenesis) that included 170 (135 unique) effector proteins (proteins functionally related to the development of a pathology) (Supplementary Table S2). IAI was defined as the inhibition of VEGF-B, PlGF, VEGF-A, Gal-1, VEGFR1, and VEGFR2.

**Figure 2:**
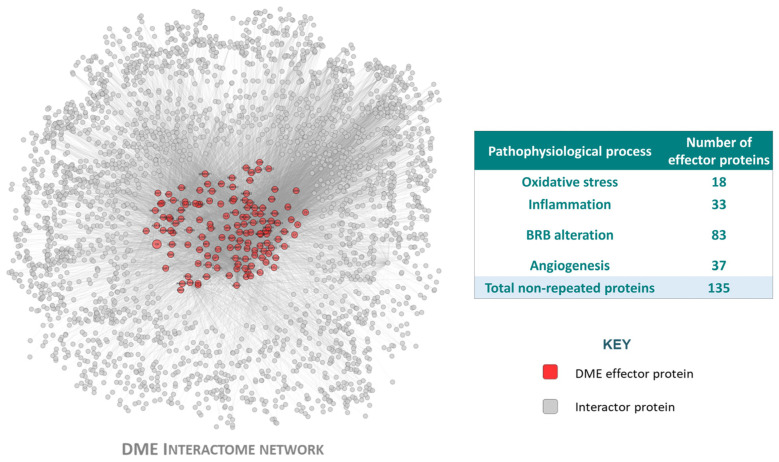
The diabetic macular edema (DME) interactome, generated from the disease characterization. Graphical representation of the DME protein network, including the effector proteins (seed protein included in the DME characterization because of its strong effect upon the disease pathophysiology, described in the compiled DME-related literature) and the interactor proteins (protein not included in the DME characterization, but interacting with at least one of the DME protein effectors) that belong to the DME pathophysiological processes (oxidative stress, inflammation, blood–retinal barrier (BRB) alteration and angiogenesis). Image created with Cytoscape v.3.9.1.

**Figure 3:**
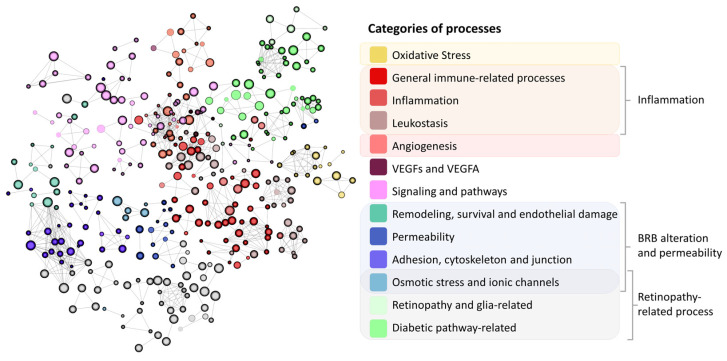
Representation of the results obtained from the enrichment analysis. The maximum Hausdorff distance between sets was established at 1.2. The size of the node is proportional to the proteins included in the represented set that were related to diabetic macular edema pathophysiological processes (oxidative stress, inflammation, blood–retinal barrier (BRB) alteration, and angiogenesis). The color of the node illustrates the overall process in which this set is involved, and the thickness of the node’s contour is inversely proportional to the false discovery rate obtained for the set. Grey color refers to unknown categories of processes. Cytoscape v.3.9.1 was used for network representation.

The DME and IAI molecular definition were integrated in the human protein–protein interaction network or human interactome (the whole set of protein interactions and functional linkages between proteins that define human physiology), rendering the condition directly connected to the human interactome (Figure 2). Based on these relationships and the rest of the human interactome, we created a TPMS-based MoA model of IAI over DME. The model was composed of 250 solutions, which comprised 2421 proteins receiving signals (predicted protein activity) from the IAI stimulus, and presented an accuracy against the training set of 93.90%. We conducted an enrichment analysis based on all model proteins with levels of predicted protein activity absolute value > 0.8 to analyze the processes modulated in the IAI’s MoA model. We observed pathways that could be broadly classified into the following categories of processes related to DME pathophysiology (Figure 3): (a) oxidative stress and hypoxia; (b) inflammation (general immune-related processes, inflammation, and leukostasis); (c) angiogenesis; (d) VEGFs and VEGF-A; (e) signaling and pathways (mitogen-activated protein kinase [MAPK], protein kinase C [PKC], nuclear factor NF-κB [NFKB], epidermal growth factor receptor [EGFR], etc.); (f) BRB alteration (remodeling, survival, and endothelial damage; permeability; adhesion cytoskeleton and junction); (g) osmotic stress and ionic channels; and (h) retinopathy-related processes (retinopathy, glia-related, and diabetic-pathway-related). The complete list of processes and their classification is provided in Supplementary Table S3.

### 2.2. Effect of IAI, VEGFR1, and VEGFR2 Signaling upon DME

After building and evaluating IAI’s in silico model to treat DME, we evaluated how the inhibition of VEGFR1 and VEGFR2 affected the expected modulation of DME. Four scenarios were considered (see Section 4.2).

**Figure 4:**
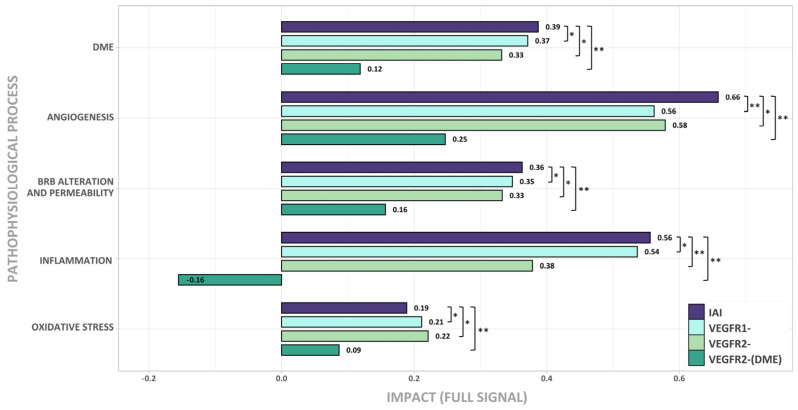
Effect of the signal inhibition of IAI, VEGFR1, VEGFR2, and VEGFR2-(DME) on DME pathophysiological processes (oxidative stress, inflammation, blood–retinal barrier (BRB) alteration, and angiogenesis). Intensity of response (Full Signal) for each scenario, according to the simulated models in diabetic macular edema (DME) pathophysiological context. IAI: inhibition of intravitreal aflibercept injection (IAI) targets (vascular endothelial growth factor A (VEGF-A), VEGF-B, placental growth factor (PlGF), and Galectin 1 (Gal-1)) and receptors VEGFR1 and VEGFR2; VEGFR1-: VEGFR1, PlGF, and VEGF-B inhibition; VEGFR2-: VEGFR2, VEGF-A, and Gal-1 inhibition; VEGFR2-(DME): VEGFR2 pathway inhibition (including VEGF-A and Gal-1 ligands) while maintaining VEGFR1 pathway activation (including VEGF-B and PlGF ligands). False discovery rate (FDR) was obtained with the Mann–Whitney U test. * FDR < 0.005 and <0.1 signal difference vs. IAI; ** FDR < 0.005 and ≥0.1 signal difference vs. IAI.

When executing the Full Signal (fSignal) evaluation (Figure 4), we found that IAI could affect all DME pathophysiological processes analyzed, with the strongest effects on angiogenesis, BRB alteration, and inflammation.

The inhibition of either the VEGFR1 or VEGFR2 signaling pathway was effective in treating DME (see DME bars in Figure 4). Interestingly, the simultaneous inhibition of both pathways increased the impact of treatment, except for oxidative stress. Angiogenesis was the process that underwent the greatest benefit from this simultaneous inhibition. Also, inhibiting either the VEGFR1 or VEGFR2 pathway generated a similar impact on oxidative stress, angiogenesis, and BRB alteration. BRB alteration was slightly more affected by the inhibition of VEGFR1, while angiogenesis and oxidative stress were slightly more impacted by the inhibition of VEGFR2. Inflammation was more relevant, in terms of its impact, on the VEGFR1 signaling pathway. The additional inhibition of the VEGFR1 signaling pathway improved the benefit of the DME therapy.

Finally, the VEGFR2-(DME) scenario showed that the activation of the VEGFR1 signaling pathway limited its effectiveness in impacting DME for all pathological processes.

The results suggest that, besides the inhibition of VEGFR2, the inhibition of the VEGFR1 pathway through IAI is crucial for the effective treatment of DME, especially in the inflammatory process.

### 2.3. Molecular Differences of IAI vs. VEGFR2-Specific Inhibition over DME

**Figure 5:**
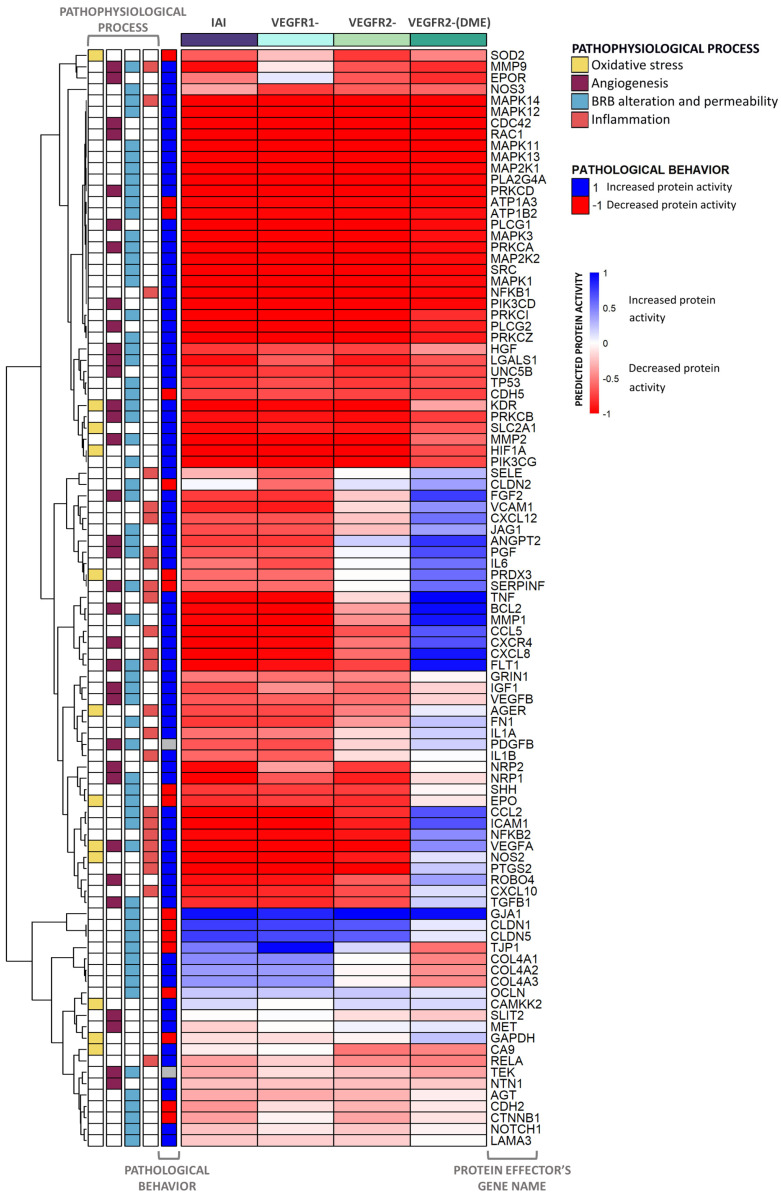
Heatmap displaying the activation of all DME protein effectors and related pathophysiological processes. The heatmap represents the average predicted protein activity induced by the different scenarios over the effector proteins of diabetic macular edema (DME), related to oxidative stress, angiogenesis, inflammation, and blood–retinal barrier (BRB) alteration, with a predicted activity ≥ |0.1| in at least one of the cohorts analyzed. The effectors are organized hierarchically, and the applied stimulus is indicated at the top of each column with the color reflecting the function. The pathological state and involvement of each effector in DME pathophysiological processes are described on the left side of the heatmap. The intensity of the color is proportional to the predicted protein activity, ranging from −1 to 1. Blue: the effector is being activated by the stimulus applied. Red: the effector is being inhibited by the stimulus applied. IAI: inhibition of intravitreal aflibercept injection (IAI) targets (vascular endothelial growth factor A (VEGF-A), VEGF-B, placental growth factor (PlGF), and Galectin 1 (Gal-1)) and receptors VEGFR1 and VEGFR2; VEGFR1-: VEGFR1, PlGF, and VEGF-B inhibition; VEGFR2-: VEGFR2, VEGF-A, and Gal-1 inhibition; VEGFR2-(DME): VEGFR2 pathway inhibition (including VEGF-A and Gal-1 ligands) while maintaining VEGFR1 pathway activation (including VEGF-B and PlGF ligands).

After evaluating the overall impact of each stimulus on DME and its pathophysiological processes, a more detailed evaluation to explore the predicted protein activity of the individual DME effectors was carried out, generating the heatmap in Figure 5. The latter was created to visualize the predicted activity of effector proteins in different populations of mathematical models. The heatmap only incorporated effectors that exhibited a minimum predicted protein activity of 0.1 in one of the cohorts.

Our data showed that the IAI treatment could modulate, and potentially reverse, the pathological state of most proteins involved in DME. The inhibition of both VEGFR1 and VEGFR2 signaling pathways regulated many protein effectors, including key effectors, such as proto-oncogene tyrosine-protein kinase Src (SRC), 1-phosphatidylinositol 4,5-bisphosphate phosphodiesterase γ (PLCG), MAPK, PKC, class I phosphoinositide 3-kinases (PI3K), NF-κB Ras-related C3 botulinum toxin substrate 1 (RAC1), and hypoxia-inducible factor 1-α (HIF1A).

The sole inhibition of VEGFR2 signaling affected the predicted activity of several effectors linked to DME pathophysiological processes, such as neuropilins (NRPs), matrix metalloproteinase-9 (MMP9), erythropoietin receptor (EPOR), neurogenic locus notch homolog protein 1 (NOTCH1), and carbonic anhydrase 9 (CA9). On the other hand, the inhibition of VEGFR1 signaling led to changes in the predicted protein activity of other proteins, including C-X-C chemokine receptors (CXCRs), FGF2, vascular cell adhesion protein 1 (VCAM1), tumor necrosis factor (TNF), apoptosis regulator Bcl-2 (BCL2), thioredoxin-dependent peroxide reductase (PRDX3), and various cytokines, permeability, and junction proteins. However, the joint inhibition of both pathways (IAI) modulated more protein effectors and did so with a greater impact than inhibiting just one of them.

Finally, the VEGFR2-(DME) scenario reversed the pathological state of a lower number of effectors (around half of the DME protein effectors) than those reversed in other scenarios, leaving some relevant effectors (e.g., interleukin-6 [IL6], FGF2, and VCAM1) in the pathological state.

**Figure 6:**
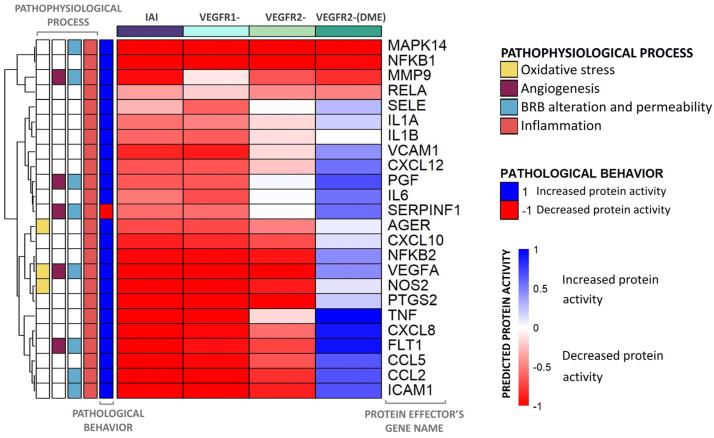
Heatmap including the predicted activity for the inflammation process proteins. The heatmap represents the average predicted protein activity induced by the different scenarios over the inflammatory effector proteins of diabetic macular edema (DME) with a predicted activity ≥ |0.1| in at least one of the cohorts analyzed. The effectors are organized hierarchically, and the applied stimulus is indicated at the top of each column, with the color reflecting the function. The pathological state and involvement of each effector in DME pathophysiological processes (oxidative stress, inflammation, blood–retinal barrier (BRB) alteration, and angiogenesis) are described on the left side of the heatmap. The intensity of the color is proportional to the predicted protein activity, ranging from −1 to 1. Blue: the effector is being activated by the stimulus applied. Red: the effector is being inhibited by the stimulus applied. IAI: inhibition of intravitreal aflibercept injection (IAI) targets (vascular endothelial growth factor A (VEGF-A), VEGF-B, placental growth factor (PlGF), and Galectin 1 (Gal-1)) and receptors VEGFR1 and VEGFR2; VEGFR1-: VEGFR1, PlGF, and VEGF-B inhibition; VEGFR2-: VEGFR2, VEGF-A, and Gal-1 inhibition; VEGFR2-(DME): VEGFR2 pathway inhibition (including VEGF-A and Gal-1 ligands) while maintaining VEGFR1 pathway activation (including VEGF-B and PlGF ligands).

According to our models, the proteins that displayed the most significant differences between scenarios belonged to the inflammation and BRB alteration processes. To further explore IAI-specific mechanisms, we focused on the inflammation process (Figure 6).

The independent inhibition of VEGFR1 and VEGFR2 signaling pathways could modulate many DME protein effectors and reverse their pathological state, including p38 (MAPK14) and NF-κB family effectors. However, the inhibition of both pathways presented the most promising results, i.e., a deeper inhibition of more effectors than any single pathway. Moreover, the modulation of E-selectin (SELE) and IL6 by inhibiting both VEGFRs also improved compared to the effect achieved through the sole inhibition of the VEGFR2 signaling.

In general, inhibiting the VEGFR1 pathway modulated more proteins within the inflammation motive of DME than inhibiting the VEGFR2 pathway. More specifically, the inhibition of VEGFR1 signaling modulated the activity of IL6, pigment epithelium-derived factor (SERPINF1), and SELE, reverting their pathophysiological state. This inhibition induced a deeper impact on the modulation of these proteins than that achieved by inhibiting the VEGFR2 pathway. On the other hand, inhibiting the VEGFR2 signaling pathway better modulated the pathological behavior of other effectors like MMP9 and transcription factor p65 (RELA). This molecular detail highlighted that the two pathways could become complementary, since each modulates protein effectors unaffected by the other pathway. All of these inflammatory effector proteins were also modulated by the IAI stimulus and included other effectors (e.g., NF-κB p100 subunit [NFKB2], VEGF-A, and nitric oxide synthase [NOS2]), but the pathological condition of SERPINF1 was not reversed by IAI and was inhibited in a DME context.

However, the inhibition of VEGFR2 in a DME context (VEGFR2-[DME] scenario) could not reverse the pathological state of some effectors, such as TNF and interleukin-8 (CXCL8).

### 2.4. Detailed IAI-Specific In Silico Molecular Mechanism

**Figure 7:**
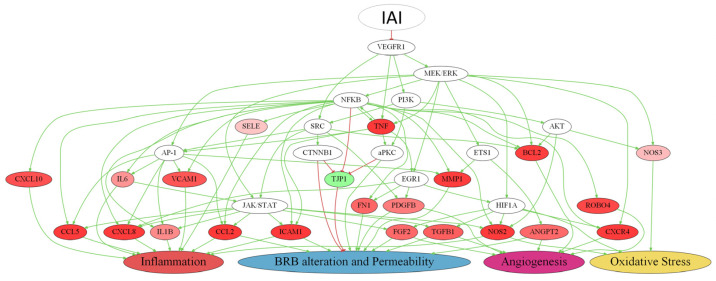
Simplified representation of the predicted IAI molecular mechanism proposed through VEGFR1. Colored protein nodes indicate the most effectively improved proteins by sustained inhibition of vascular endothelial growth factor 1 (VEGFR1) through intravitreal aflibercept injection (IAI) treatment in diabetic macular edema (DME) pathophysiological processes (inflammation, blood–retinal barrier (BRB) alteration, angiogenesis, and oxidative stress), where green means activation and red implies inhibition. The modulation range is from −1 (inhibition) to +1 (activation), and the more affected a protein is, the darker its color. Each effector is connected to the motive that it influences based on its predicted activity, either advancing or slowing down the growth of the pathophysiological process related to DME. The figure was created with Graphviz software v.2.38 https://graphviz.gitlab.io/ (accessed on 4 November 2022). The complete scheme is available in Supplementary Figure S1.

Notwithstanding the multi-target nature of IAI, its MoA was analyzed considering solely the VEGFR1 signaling pathway to obtain a proper representation (Figure 7, Supplementary Figure S1 and Table S4). In this context, the VEGFR1 inhibition had the most significant impact on two pathophysiological processes: inflammation (10 effector proteins) and BRB alteration (11 effector proteins) (Supplementary Table S2). In addition, VEGFR1 also played a role in the modulation of oxidative stress (three effector proteins) and angiogenesis (seven effector proteins), albeit with a minor impact (Supplementary Table S2). In the scheme, the molecule with the most interactions was the pro-inflammatory trigger protein NF-κB, followed by intracellular signaling proteins of cell survival MEK/ERK, JAK/STAT, and pro-angiogenic trigger factor HIF1A. The molecules that stood out were those modulated by the VEGFR1 pathway (i.e., TNF, C-C motif chemokine 5 [CCL5], CXCL8, CCL2, intercellular adhesion molecule 1 [ICAM1], interstitial collagenase [MMP1], NOS2, and tight junction protein ZO-1 [TJP1]). All of these pro-inflammatory molecules seemed highly interconnected and were inhibited by IAI, except for TJP1 (a gene with permeability-related activity), which was activated.

## 3. Discussion

The present approach developed an objective IAI’s MoA model, including the main known pathways of DME, i.e., angiogenesis, inflammation, oxidative stress, BRB alteration, and diabetes-related features. A molecular analysis was undertaken to assess the advantages of IAI’s multi-target nature and control over VEGFR1 activation in the context of DME. Our model showed that, while both VEGFRs’ signaling pathways have therapeutic potential, the joint inhibition of VEGFR1 and VEGFR2 signaling pathways by IAI may result in more effective action against the pathological changes originated by DME. We further explored the benefit of the specific VEGFR1 inhibition, highlighting specific proteins and pathways, especially those involved in inflammation.

In our modeling approach, IAI exerted its effects on the signaling pathways activated by VEGF-A, VEGF-B, PlGF, and Gal-1 by interacting with their corresponding receptors. The present mathematical conceptualization was aligned with the existing body of evidence. Experiments have demonstrated that IAI has a stronger affinity for VEGF-A than its natural receptors, and that it could also effectively bind and induce the inhibition of PlGF, Gal-1, and VEGF-B [11,33,34]. PlGF has been related to angiogenesis, inflammation, and BRB alteration, while the overexpression of both VEGF-A and VEGF-B has been linked to the breakdown of BRB and retinal angiogenesis events [35,36,37]. On the other hand, Gal-1 contributes to the inflammation processes and angiogenesis in DME [38]. Therefore, the inhibition of all four target molecules by IAI supported its efficacy against DME.

Our data displaying the activation of all DME protein effectors showed that VEGFR1 inhibition led to an enhanced modulation of several key effector proteins when compared with VEGFR2 inhibition. These effectors are involved in DME’s main pathophysiology events, including angiogenesis (PDGFB, BCL2, C-X-C motif chemokine receptor 4 [CXCR4], FGF2, roundabout homolog 4 [ROBO4]), inflammation (ICAM1, CCL5, interleukin-1β [IL1B], CCL2, VCAM1, SELE, C-X-C motif chemokine ligand 10 [CXCL10], IL6, CXCL8, TNF-α), oxidative stress (NOS2, NOS3), and BRB alteration (transforming growth factor β-1 proprotein [TGFB], MMP1, TJP1, fibronectin [FN1], and ANGPT2). A high proportion, more than 90%, of these analyzed inflammatory-related proteins were modulated by IAI, which reinforced its potential as a first-line therapeutic option for the pharmacological treatment of DME. Concretely, VEGFR1 has been shown to be associated with vascular development [39], vascular leakage [40,41], inflammatory processes [40,42,43,44,45,46], angiogenesis [14], oxidative stress, and BRB alteration [47] in human and mouse models, in accordance with the predictions of our models. Therefore, the potential inhibition of the involved signaling pathways could contribute toward a more effective treatment of DME.

The heatmap results showed that the modulation of both the VEGFR1 and VEGFR2 pathways had a greater impact on the predicted proteins, with decreased activity, than the modulation of the VEGFR2 pathway alone. This could be explained by the more effective modulation of all of the involved pathophysiological mechanisms when more pathways were affected. Furthermore, most predicted proteins that showed decreased activation in VEGFR1 belong to the inflammatory group. This particular observation is significant, since the inflammatory environment is increasingly recognized as a crucial factor in the development and severity of DME [48]. The aqueous and vitreous levels of pro-inflammatory and pro-angiogenic cytokines are more elevated in eyes with DME than in those of diabetic patients without retinopathy and those of control groups [49,50,51,52]. Furthermore, these cytokine levels have been related to DME severity, increased retinal thickness measured by optical coherence tomography, and the presence of leakage measured by fluorescein angiography [50,51]. Notably, the increased levels of aqueous VEGF, monocyte chemoattractant protein 1 (MCP-1), IL6, and TNF-α have been identified as main predictors of DME [53]. The findings from our model, displayed as heatmaps, suggested that the predicted impact of IAI significantly reduced the activity of all of the aforementioned molecules. Since these molecules have been correlated with the emergence and severity of DME [50,51,52,53], they are also likely involved in IAI’s MoA.

Regarding the VEGFR1 pathway, several other inflammation-related proteins displayed a predicted decrease in activity after IAI treatment. Among them was NOS2, which has been implicated in DME by increasing the enzymatic activity in the retina, particularly in Müller cells [54]. The overexpression of nitric oxide by NOS2 has also been linked to increased vascular permeability, inflammation, and oxidative stress, all of which contribute to the development and progression of DME [55]. On the other hand, the NF-κB pathway has been demonstrated to affect multiple genes involved in inflammation, angiogenesis, and oxidative stress [56], including the NOS2 effector protein [57]. These findings are in line with the predicted function of the NF-κB family in our model, which was downregulated by IAI in the VEGFR1 pathway. According to our model, this downregulation could exert multiple actions that decrease the oxidative status and inflammatory levels. This fact is in line with the role of the NF-κB family in oxidative stress and inflammation pointed out in the literature [58,59]. TNF-α was another downstream effector protein predicted to decrease with the inhibition of the VEGFR1 signaling pathway. As previously mentioned, TNF-α has been shown to be elevated in the vitreous fluid of DME patients [60]. Beyond its direct effects on NOS2, TNF-α can also activate NF-κB signaling, contributing to inflammation, angiogenesis, and oxidative stress events [61]. Therefore, and as expected, in our model, an inhibition of TNF-α expression decreased the activity of the NF-κB pathway and NOS2. Taking all of these factors together, according to our model predictions, a sustained inactivation of the VEGFR1 pathway by IAI could lead to a significant reduction in the inflammation and oxidative stress events/processes.

VEGFR1 and VEGFR2 pathways have also been proven to be involved in BRB alteration [62], in agreement with our results. VEGFR1 leads to signaling mainly mediated through the effector protein p38 [63]. The increase in BRB alteration through the VEGFR2 pathway can be achieved by several signaling pathways, including the renin–angiotensin system, PI3K/Akt, or phospholipase C (PLC)γ [64]. According to our mathematical model, inhibiting both VEGFR1 and VEGFR2 could potentially contribute to the protective effects of IAI against BRB dysfunction and alteration in DME.

Notably, some cellular types principally express VEGFR1 and others VEGFR2, such as the retinal capillary endothelial cells for VEGFR2 [10]. VEGFR1 expression has been detected predominantly in monocytes and macrophages [65], but also in retinal and choroidal epithelial and endothelial cells [66,67], retinal vessel pericytes [66], Müller cells [68], photoreceptor cells [69], and retinal pigment epithelial cells [69]. This differential pattern of expression implies a differential cellular effect. Several studies have demonstrated the role of VEGFR1 in inflammatory processes, involving monocytes and macrophages, among other components of innate immunity [42,70,71,72]. Thus, blocking VEGFR1 should have a direct impact on DME, causing an inflammatory decrease and, consequently, lower disease activity. Importantly, the results of our enrichment analysis showed that some of the processes found to be enriched (e.g., cell aging, homeostasis of the number of cells, and regulation of autophagy) did not belong to the pathophysiological processes defined by the characterization. This would demonstrate that our model was able to find results beyond the characterization and would indicate that the IAI’s MoA could affect other areas of DME that are less known. This finding would imply a relationship between inflammation and the disease’s chronicity and/or severity. Concretely, the sustained inhibition of VEGFR1 signaling could explain the better visual acuity outcomes in patients treated with IAI than that of patients receiving other anti-VEGF treatments in randomized clinical trials [21,73,74,75,76,77].

By contrast, VEGFR2 is mainly expressed in capillary endothelial cells [78] and has been shown to have an outstanding role in angiogenesis and vascular permeability, mainly mediated by its ligand VEGFA [62]. However, the VEGFR2 signaling pathway also leads to the upregulation of several pro-inflammatory cytokines, such as MCP-1 and ICAM-1 via NF-κB [79,80,81], which could partly explain the more effective impact of VEGFR1 on inflammation.

The present work involves modeling the MoA of IAI in the context of DME, which aids understanding of the underlying therapeutic potential of this drug widely demonstrated in randomized clinical trials [20,21,22]. We have intentionally focused the discussion on VEGFR1 since the effects of IAI on VEGFR2 are well known.

Of note, our results regarding IAI’s activity were correlated with clinical practice, since IAI is currently used as first-line treatment in DME patients with the worst vision. For instance, the guidelines for the management of DME by the European Society of Retina Specialists recommend IAI as the drug of choice in DME eyes with baseline best-corrected visual acuity below 69 letters because of its proven superiority to other DME treatments [82].

The TPMS technology has been previously used to shed light on the complex MoA of certain drugs, such as the immunomodulatory mechanisms of eltrombopag [83], or the cardiovascular effects of the anti-diabetic empaglifozin [31]. This technology can be applied to achieve other medically or biologically challenging objectives, such as identifying the optimal patient profile for treatment with a drug. Besides, TPMS technology has been used, in combination with microarray and miRNA data, to unveil potential mechanisms behind the lack of response to corticosteroids in ulcerative colitis patients [29]. In addition, this in silico technique has successfully differentiated the patients who would benefit the most from different attention-deficit/hyperactivity disorder treatments in combination with other modeling techniques to obtain quantitative systems pharmacology (QSP)-based models [84,85]. Our objective in this study was to understand how IAI’s target profile, including the VEGFR1 axis modulation, operated to treat DME, as a first step towards the in vitro, in vivo, or clinical evaluation of our findings. However, some patients have been reported to present persistent DME after IAI treatment [74,86]. While recent prospective and real-world studies have been undertaken to identify disease markers and factors related to reduced response [87,88], the proposed models herein could be enhanced in the future to address the molecular reasons behind non-responsiveness in some DME patients. Similarly, these models could serve as the foundation for more complex QSP models, which would allow comparing different dosing schemes in mechanistical terms [89].

### Study Limitations

The current study suffers from several limitations that must be considered. First, the study was based on existing literature and, therefore, could have been biased toward processes that have been widely reported in the past and information annotated in databases. To address this, we evaluated the network around the key targets of the study—VEGFR1, VEGFR2, VEGFA, VEGFB, PlGF, and Gal-1—and explored the available bibliography to ensure that the main reported functions and interactions were included in our model, particularly in the context of DME. Nevertheless, these sampling-methods-based models were built by considering the whole human protein network and a wide range of drug-pathology relationships (Supplementary Table S6), not only limited to DME or ophthalmological or inflammatory conditions, and they present accuracies above 90% in models. Second, given the complexity of the VEGFA interaction with VEGFR1 and VEGFR2, in this study, we assumed that VEGFA–VEGFR1 signaling was irrelevant since it is limited by VEGFR1 solubility and the presence of VEGFB or PlGF. Notably, our VEGFR2 results cannot be used to directly infer conclusions regarding VEGFA inhibition (the mechanism of ranibizumab or bevacizumab, for instance), because its interaction with VEGFR1 and VEGFR2 is complex [10]. VEGFB and PlGF present a higher affinity for VEGFR1, while the signaling of VEGFR1 by VEGFA is not preferential and depends on the dissociation constant of VEGFR1 and the solubility of VEGFB or PlGF [14]. Considering this, we could speculate that the blockade of all ligands, rather than just VEGFA, would result in greater inhibitory action on the signaling pathway mediated by VEGFR1 and VEGFR2. Third, oxidative stress signaling events are largely dependent on non-protein species [90], and, hence, the results of a protein-based model might not reflect the full picture. Fourth, our study of the effect of IAI was limited to the processes defined during the disease characterization, in which we adopted a conservative approach, including processes well established to induce the accumulation of intraretinal fluid. With this approach we have overlooked other processes involved in retinopathy that could also be relevant in the process. For instance, neurodegeneration has been reported to occur earlier than and independently of vascular damage [91]. However, since the implication of neurodegeneration on vascular regulation remains unclear [92,93], we did not consider “neurodegeneration” a causal process. Rather, we considered other processes that could have a more clear role in DME, such as oxidative stress generation and inflammation. Thus, importantly, conclusions are susceptible of being updated over time if new developments in the pathophysiology of DME are described, or if prospective data and new information are generated (e.g., specific molecular data on IAI treatment of DME), making them more accurate regarding DME pathophysiology. Moreover, although this in silico modeling allowed us to obtain more insight into the IAI molecular mechanism in the pathophysiology of DME beyond its antiangiogenic effect, which could contribute toward guiding further research, the results obtained in this work must be validated or refuted through the performance of in vitro and/or in vivo experimental studies. Finally, it is important to note that in silico models reflect protein activity actions, and the results may not translate directly into protein expression.

## 4. Materials and Methods

We applied the Therapeutic Performance Mapping System (TPMS), a proprietary technology of Anaxomics (Barcelona, Spain) [30], to analyze the impact and the MoA of IAI on DME. The TPMS is a systems-biology-based machine learning approach that creates multiple models to understand the MoA of a drug. We obtained the results through a strategy that involved collecting information about DME and the drug of study, followed by analyzing the model outputs in terms of the parameters explained above (Figure 1). We carried out our analysis focusing on IAI targets and the signaling pathways controlled by these targets through their receptors VEGFR1 and VEGFR2.

### 4.1. Drug and Disease Characterization

We conducted the molecular characterization of DME through a meticulous manual curation of the related scientific literature on the subject. We searched the PubMed database [94] on 23 December 2020 for reviews published in the last 13 years on the molecular pathogenesis and pathophysiology of DME. The search query consisted of the terms “diabetic macular edema”[Title] OR “diabetic macular oedema”[Title] OR “DME”[Title] AND (“molecular”[Title/Abstract] OR “pathophysiology”[Title/Abstract] OR “pathogenesis”[Title/Abstract]), which returned 146 results. We analyzed the identified publications at the title and abstract level, aiming to identify the central pathophysiological processes involved in DME (Figure 2). The corresponding molecular protein effectors playing a biological role in these processes (Supplementary Table S2) were also retrieved from this bibliography review. Relevant articles were identified through the reference lists of the initial set of articles when a reference to protein effector candidates was made. When a protein candidate was detected in the compiled literature, it was included as disease effector if the reference contained or cited functional evidence of the role of a protein activity change in the causal development of the pathology. This procedure avoided including proteins that might be a marker or reflection of the pathology rather than a causal effector. When the reported evidence for a protein’s role in disease was deemed insufficient from a functional point of view to be assigned as a disease effector, additional PubMed searches focused on this protein were performed, spanning all known protein name synonyms as per UniProtKB codes.

To gain a comprehensive understanding of the characteristics of IAI and its MoA, we thoroughly reviewed official documents related to the drug, including those from the EMA [95], the FDA [96], and its product monographs. Additionally, we sourced target information from specialized databases [97,98,99]. Lastly, we reviewed recent publications in PubMed on 2 February 2021 to identify the known targets and other mechanistic details currently documented regarding the drug (Supplementary Table S5). The specific search consisted of the following: (Aflibercept[Title/Abstract] OR eylea[Title/Abstract] OR zaltrap[Title/Abstract] OR ziv-aflibercept[Title/Abstract]) AND (molecular[Title/Abstract] OR mechanism[Title/Abstract] OR target[Title/Abstract] OR effect[Title/Abstract] OR action[Title/Abstract]) AND (“diabetic macular edema”[Title/Abstract] OR “DME”[Title/Abstract] OR “diabetic macular edema”[Title/Abstract] OR “macular edema”[Title/Abstract]), retrieving 84 results from the last 5 years. We used all known names for aflibercept in the search to increase article matches, but upon article review, we only included information contextualized to the ocular indication or generally applying to IAI’s MoA.

### 4.2. Modeling: Therapeutic Performance Mapping System and Sampling Methods

The TPMS technique integrates available biological, pharmacological, and medical knowledge to simulate human physiology in silico [30,100,101]. The methodology employed has been previously described [30,32,102]. Briefly, the TPMS uses machine learning and pattern recognition techniques to generate mathematical models that describe the relationship between a stimulus (the modulation of VEGFRs through its targets by IAI) and response (DME and its pathophysiological processes). This systems biology modeling approach starts off a human protein–protein interaction network, including not only physical, but also regulatory and functional relationships among proteins and genes (Supplementary Table S6) [30,32]. The models are generated using an algorithm similar to a Multilayer Perceptron of an Artificial Neural Network over the human protein–protein interaction network (where neurons/nodes are the proteins and the edges of the network are used to transfer the information) [30], and are trained using biological and clinical data (Supplementary Table S6). Then, a stimulus is applied to the node corresponding to the drug target under study, and the signal traverses the network until it reaches the response nodes, which consist of the genes/proteins involved in the phenotype of interest. To accommodate the complexity of biological systems and the likelihood of multiple pathways, nodes are assigned weights, based on training with an extensive drug indication database, that modulate signal flow. The algorithm iteratively repeats multiple times, and the final MoA represents a compilation of the most probable functional models build on the extraction of common patterns across all obtained solutions. We obtain an optimized network of proteins/genes interconnected based on probable functional relationships relevant for explaining the phenotype of interest, rather than a classification/regression prediction score, aimed to explain a known biological effect and its restrictions instead of predicting its occurrence. This methodology allows functional properties and mechanistic insights to be revealed, suggesting new hypotheses for testing in vitro or in vivo, and it is fully described in Jorba et al. [30].

**Figure 8:**
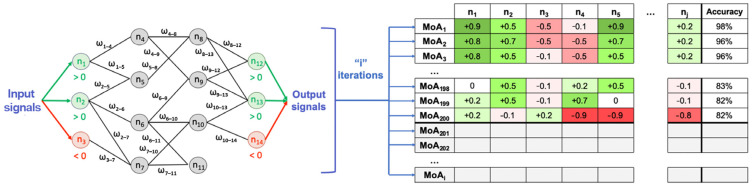
Schematic example on how the Therapeutic Performance Mapping System (TPMS) is applied to weight (ω) the links to determine the mechanism of action (MoA) of a drug. Simplified example of the method over a simplified network, transmitting information over the human protein–protein interaction network using a Multilayer Perceptron-like algorithm. After a given number of iterations (i), we obtain a collection of MoAs. Rows represent the MoAs and columns the output signal values of the proteins (n, nodes of the network). The intensity of the color ranging from < 0 (red) to > 0 (green). The final column shows the accuracy of the model as a percentage of the number restrictions accomplished. In this simplified example, $n_{5}$ is linked to $n_{1}$ and $n_{2}$. The output signal of $n_{5}$ is $n_{5}=\tanh\left(n_{1}\cdot \omega_{1-5}+n_{2}\cdot \omega_{2-5}\right)$. Figure obtained from Jorba et al. [30] with slight modifications.

Briefly, the models consist of all plausible MoA solutions considering the network and training set information, and are composed of protein paths linking the stimulus and response. Each link between the proteins can take a value of either 1 (activating interaction) or −1 (inhibiting interaction), and is assigned a weight ($\omega_{l}$), i.e., the parameters to solve according to the training set information. Each node (protein) of the human protein–protein interaction network receives, as input, the output of the incoming connected nodes, weighted by each link weight. The sum of inputs is transformed by a hyperbolic tangent function to generate the score of the node and becomes the output signal of the node towards outgoing nodes (Figure 8) [30]. The $\omega_{l}$ parameters are obtained by optimization using a Stochastic Optimization Method based on Simulated Annealing [103]. Weight adjustment is performed by running the simulation using the stimulus and response sets of known drug–medical condition relationships. This information is derived from a database that contains publicly available information about drugs and all known indications and side effects, called training set for reference (Supplementary Table S6). In this sense, edges are attributed a higher value if they lead to a biological interpretation known to be true in the training set and comply with the restrictions applied to the specific model. In other words, in absolute terms, nodes are able to pass more signal through if they are frequently used to provoke the known effect of a drug over a medical condition. This procedure maximizes the percentage of satisfied biological knowledge in the database, thus maximizing accuracy. Thus, the values of the effector proteins are the closest to their expected values and are always adjusted to the training set considering both positive and negative stimulus response relationships (Supplementary Table S6). The accuracy of the models is determined by the percentage of fulfillment of restrictions in the training set. The number of $\omega_{l}$ parameters can be very high and the size of the training set is usually not enough to find a unique solution. For that reason, many plausible final models can be obtained (Figure 8) [30].

Specifically, to define the model, we considered IAI an inhibitory stimulus targeting the four targets and the VEGFR1 and VEGFR2 receptors and considered DME (Supplementary Table S2) as the response. The final optimized model consisted of 250 model solutions explaining plausible relationships between IAI and the pathophysiological mechanism of DME [30].

Once a mechanistic model is generated, it can be used to calculate how the signal flows through the network from different stimuli to the response and thereby to calculate the predicted protein activity (ranging from −1 [completely inhibited] to 1 [completely activated]) of any protein involved in the model. Therefore, once the IAI mechanistic model over DME was established, the following scenarios (drug mechanisms) were applied in the DME model: (i) IAI: Inhibition of IAI targets (VEGF-B, PlGF, VEGF-A, Gal-1) and VEGFR1 and VEGFR2, (ii) VEGFR1-: Inhibition of VEGFR1, VEGF-B, and PlGF ligands [impact of the inhibition of VEGFR1 pathway], (iii) VEGFR2-: Inhibition of VEGFR2, VEGF-A, and Gal-1 ligands [impact of the inhibition of VEGFR2 pathway], and (iv) VEGFR2-(DME): Inhibition of VEGFR2 pathway, including VEGF-A and Gal-1 ligands, while maintaining VEGFR1 activation, including VEGF-B and PlGF [impact of the inhibition of VEGFR2 pathway in the context of DME, i.e., with VEGFR1 pathway activated]. The VEGFR2-(DME) scenario was explored specifically to account for the downstream interlocking of VEGFR1- vs. VEGFR2-mediated processes [13] to properly explore a situation in which VEGFR2 alone is inhibited while VEGFR1 is consistently activated, as in DME, where its ligands are increased [104].

The impact of treatments on the disease processes based on the predicted protein activity of the effectors defined for each pathophysiological process or disease was measured through the predicted protein-activity-derived fSignal. The fSignal (ranging from −1 to 1) represents the average predicted protein activity received by the entire set of response proteins. This measurement is obtained by evaluating the signal values of all response proteins in response to the stimulation of the model and computing the mean value, following the equation below:

(1) $$fSignal=\frac{1}{n}\sum_{i=1}^{n}w_{i}o_{i}$$

The fSignal equation is derived from the TSignal [30] and includes the following terms: *n* applies to the number of modulated proteins within the set; *i* refers to the response protein; *w_i_* refers to the sign of response of protein *i* according to the response set definition; and *o_i_* is the signal value achieved by response protein *i* after stimulating the system. This model analysis offered a molecular point of view on the drug–pathophysiology relationship and how the treatments (stimuli) could modulate the pathophysiology.

Although the TPMS models are protein-based, the interactome in which they are built includes gene and RNA regulation data. Therefore, for standardization purposes, we used gene names to refer to all genes/proteins mentioned in this manuscript when referring to model results. To facilitate the reading, we provide further information on the genes’/proteins’ nomenclature in Supplementary Table S1.

### 4.3. Analysis

#### 4.3.1. Enrichment Analysis

From the predicted protein activity values of all model proteins, we made a hypergeometric enrichment analysis to explore the processes modulated by the MoA of IAI [105]. The analysis included the proteins with “|*predicted protein activity*|” greater than 0.8 and used the complete list of proteins to be included in the model as a reference list for the enrichment analysis. We ran the enrichment considering the Gene Ontology (GO) Biological Processes as defined by the UniProtKB database [106] and set the false discovery rate (FDR), which measures the rate of type I error (incorrectly rejected null hypotheses) in a list of rejected hypotheses, at q-value > 0.001. We discarded weakly represented or non-specific GO terms (>10 or <200/300 genes) from the results. We calculated the distance between the enriched sets according to the protein network by applying the Hausdorff distance (which measures the similarity between two subsets of a metric space, with a smaller distance indicating more protein coincidences/similarities) for interpretation and visualization purposes, following the equation below [107]:

(2) $$d_{H}\left(X,Y\right)=max\left\{\;\begin{array}{c} \begin{array}{c} sup\\ x\in X \end{array}\;\begin{array}{c} inf\\ y\in Y \end{array}\;\begin{array}{c} d\left(x,y\right), \end{array} \end{array}\begin{array}{c} \begin{array}{c} sup\\ y\in Y \end{array}\;\begin{array}{c} inf\\ x\in X \end{array}\;\begin{array}{c} d\left(x,y\right) \end{array} \end{array}\;\;\right\}$$

Network representation was performed using Cytoscape v.3.9.1 [108].

#### 4.3.2. Evaluation of Predicted Protein Activity per VEGFR: Heatmaps

We generated heatmaps to visualize the effectors’ predicted protein activity in different scenarios of mathematical models. We used the R package pheatmap (version 1.0.12) running on the R version 4.2.2 software (R Development Core Team, 2022). The heatmap only incorporated effectors that exhibited a minimum predicted protein activity of 0.1 in one of the cohorts. We organized the effectors hierarchically.

#### 4.3.3. Multi-Target Mechanism of Action and Representation

Considering the multi-target nature of IAI, we sought to find the best approach to representing its signals. To focus on the role of the VEGFR1 signaling pathway modulation on the IAI-specific MoA, we selected DME effectors that were more deeply modulated and reverted their pathological status due to VEGFR1 inhibition. Additionally, we further filtered the effectors to prioritize those with a difference in predicted protein activity of at least 0.1 between that induced by VEGFR1 inhibition and VEGFR2 inhibition individually, and whose pathological behavior was reverted when treated with IAI. We evaluated the paths leading to these effectors and selected the ones fulfilling the following criteria: (a) including VEGFR1 inhibition; and (b) minimum frequency of 75% in the pool of 250 solutions conforming to the model. We manually confirmed all of the links in the evaluated paths by checking the database and literature. We used GraphViz version 2.38 to render the representations.

## 5. Conclusions

These in silico models, obtained with the TPMS technology, successfully replicated the main pathophysiological events related to DME. The models allowed us to explore the MoA of IAI, highlighting the implication of the VEGFR1 and VEGFR2 signaling pathways on the disease. The results indicated that IAI could impact inflammation, oxidative stress, angiogenesis, and BRB alteration, all of which are pathophysiological processes involved in the progression and severity of DME. Interestingly, although the sustained VEGFR1 blockage in IAI’s MoA appeared to improve the treatment impact over most DME pathophysiological processes, it could have especially relevant therapeutic potential in the inflammatory process. This work contributes toward explaining the therapeutic effect of IAI on DME, proposing detailed molecular mechanisms beyond the direct effect upon its targets, and may pave the way for a new approach to analyzing DME’s pathophysiology, highlighting the important factors for its treatment. Nonetheless, considering the theoretical nature of this work, in vitro validation assays are necessary to confirm the hypotheses and corroborate IAI’s real-life clinical results.

## Data Availability

The data presented in this study are available in the Supplementary Materials; further inquiries can be directed to the corresponding author.

## Supplementary Materials

The following supporting information can be downloaded at: https://www.mdpi.com/article/10.3390/ijms25073621/s1.
