# Supplementary material for: Aflibercept Off-Target Effects in Diabetic Macular Edema: An In Silico Modeling Approach
Source: Int J Mol Sci. 2024 Mar 23;25(7):3621. doi: 10.3390/ijms25073621 (PMC11011561; doi:10.3390/ijms25073621)
Supplement: Supplementary file 1 [file ijms-25-03621-s001.zip › Supplementary Figure S1.pdf]

Supplementary Figure S1. Representation of Therapeutic Performance Mapping System (TPMS) technology-predicted intravitreal aflibercept injection (IAI) mechanism through vascular endothelial growth factor receptor 1 (VEGFR1). Protein nodes indicate the most effectively improved proteins by sustained inhibition of VEGFR1 through IAI treatment, where green arrows imply a relationship of activation and red arrows a relationship of inhibition. The number on each link indicates the number of the corresponding entry on Supplementary Table S5, to retrieve the sources of information found in the scientific literature supporting the predicted mechanisms. This figure is the detailed version of Figure 7 in the main text. This figure was created using Graphviz software (https://graphviz.gitlab.io/) v.2.38.
