# Supplementary figures and images for: Aflibercept Off-Target Effects in Diabetic Macular Edema: An In Silico Modeling Approach
Source: Int J Mol Sci. 2024 Mar 23;25(7):3621. doi: 10.3390/ijms25073621 (PMC11011561; doi:10.3390/ijms25073621)

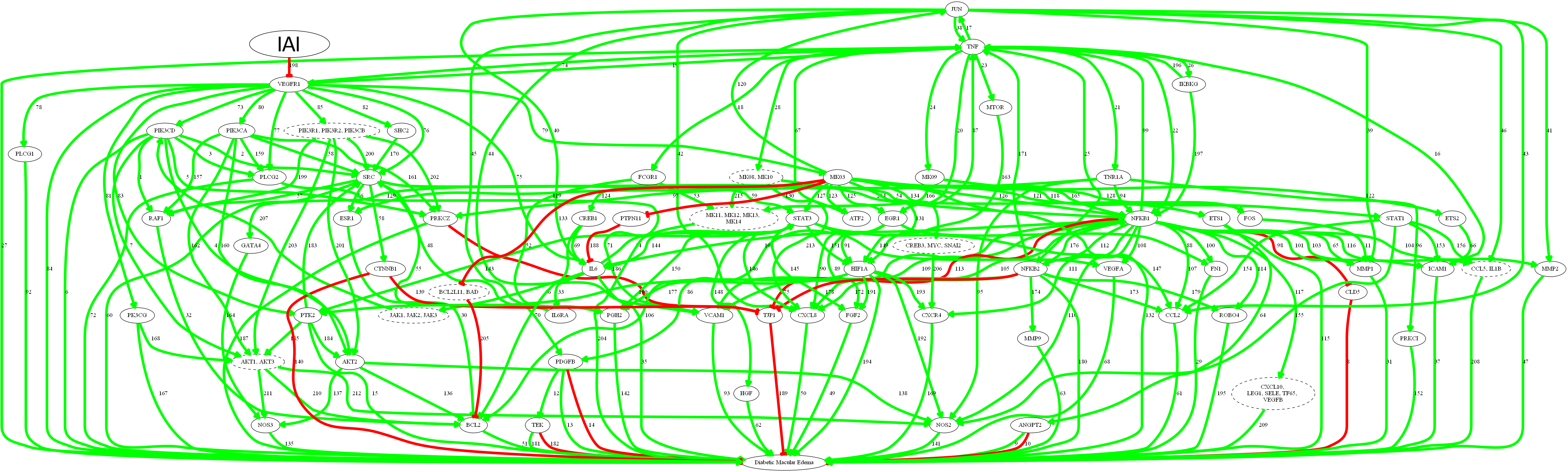

Supplement: Supplementary file 1 [file ijms-25-03621-s001.zip › Figure S1.tif]
